# Unrevealing model compounds of soil conditioners impacts on the wheat straw autohydrolysis efficiency and enzymatic hydrolysis

**DOI:** 10.1186/s13068-020-01763-3

**Published:** 2020-07-13

**Authors:** Xinxing Wu, Wei Tang, Chen Huang, Caoxing Huang, Chenhuan Lai, Qiang Yong

**Affiliations:** 1 Key Laboratory of Forestry Genetics & Biotechnology (Nanjing Forestry University), Ministry of Education, Nanjing, 210037 People’s Republic of China; 2 Jiangsu Co-Innovation Center of Efficient Processing and Utilization of Forest Resources, College of Chemical Engineering, Nanjing Forestry University, Nanjing, 210037 People’s Republic of China

**Keywords:** Autohydrolysis, Phosphate, Humate, Enzymatic hydrolysis, Enzymatic accessibility

## Abstract

**Background:**

Soil-derived exogenous ash (EA) poses a challenge toward lignocellulosic autohydrolysis due to its buffering capacity. Previous works focusing on this phenomenon have failed to also investigate the role that soluble salts, and organic matter plays in this system. Herein, sodium phosphate and sodium humate were employed as model buffering compounds representing soluble salts and organic matter and dosed into a de-ashed wheat straw (DWS) autohydrolysis process to show the potential impacts of WS attached soil conditioners on the WS autohydrolysis efficiency which would further affect the enzymatic digestibility of autohydrolyzed WS.

**Results:**

Results showed that with the increasing loadings of sodium phosphate and sodium humate resulted in elevated pH values (from 4.0 to 5.1 and from 4.1 to 4.7, respectively). Meanwhile, the reductions of xylan removal yields from ~ 84.3–61.4% to 72.3–53.0% by loading (1–30 g/L) sodium phosphate and sodium humate during WS autohydrolysis lead to a significant decrease of cellulose accessibilities which finally lead to a reduction of the enzymatic digestibility of autohydrolyzed WS from ~ 75.4–77.2% to 47.3–57.7%.

**Conclusion:**

The existence of different types soil conditioner model compounds results in various component fractions from autohydrolyzed WS in the process of autohydrolysis. A lack of sufficient xylan removal was found to drive the significant decrease in enzymatic accessibility. The results demonstrated the various effects of two typical tested soil conditioners on WS autohydrolysis and enzymatic hydrolysis.

## Background

With the developments of modern agriculture, approximately 200 billion tons of agricultural residues are now generated annually across the world [1]. Effective utilization of such huge quantities of lignocellulosic materials has become a key goal for societies that find themselves with such an overabundance [2]. One promising avenue for these materials was to use them as feedstock for producing bioethanol, amongst other chemicals. Doing so could both reduce greenhouse gases emissions and promote carbon balance in ecosystem [3–5]. Due to its abundant benefits, many researchers attempted to advance the utilization of lignocellulose or lignocellulose-based products [6–8]. One particularly abundant agricultural residue is wheat straw, which is considered an interesting feedstock for biorefining due to its high carbohydrate content (~ 28–39 wt% cellulose and ~ 23–24 wt% hemicellulose). However, efficient utilization of wheat straw to generate valuable products requires a both efficient and affordable pretreatment stage in order for subsequent enzymatic hydrolysis to render acceptable quantities of upgradable monosaccharides [9].

To date, an extensive breadth of pretreatment methods have been developed to disrupt the solid matrix comprising lignocellulosic biomasses [10–13]. Autohydrolysis, which employs water as the only media, remains a potential prospect for industrial application due to its simplicity, low cost, and efficiency. It has also been reported to pair well with standard cellulolytic hydrolysis unit operations [14]. Over the course of autohydrolysis pretreatment, significant amounts of hemicellulose plus a minor amount of lignin are degraded and rendered soluble in the aqueous media. To achieve this effect, autoionized water at high temperatures leads to abscission of hemicellulosic acetyl groups which render the aqueous medium mildly acidic (pH 3–4). However, this causes autohydrolysis to be a pH-dependent pretreatment. Therefore, fluctuations to pretreatment efficiency tend to be highly related to the raw material properties, including hemicellulosic acetyl content and exogenic ash (“EA”).

Imprecise harvesting and handling of agricultural residues inevitably results in the inclusion of farmland soil within feedstock. The EA content in biomass after harvesting could exceed 20 wt% of the dry weight of biomass by multi-pass operations [15]. Previously, some research has noted that excessive ash found in agriculture residues results in the autohydrolysis medium exhibiting strong buffer capacity and therefore low autohydrolysis efficiency which could finally lead to reductions of enzymatic hydrolysis efficiency. Due to this effect, a water-washing stage is often applied to reduce the ash content of agricultural residues in order to achieve the desired level of autohydrolysis efficiency [16]. The mechanism for this buffering was recently found to be cation exchange reactions caused by insoluble minerals (ash) present in the soil [17]. However, the buffering caused by two other key fractions of EA, soluble salts and organic matter, are overlooked due to their relatively minor quantities and the amounts of the two residual components in EA would be enhanced by fertilization of soil.

Growth and proliferation of agricultural crops tends to be highly dependent upon the presence of phosphorus provided by fertilizers widely used in cultivation of agricultural crops [18]. The utilization of phosphate fertilizers could accelerate the growth of crop roots, promote the absorption of water and nutrients by crops, improve the water use efficiency of crops and the ability to absorb short-term drought during water shortage [19]. Besides, phosphate, as the main component of phosphate fertilizers, has the ability to combine with metal cations in soil to increase the soil buffering capacity, which can behave as a pH buffering compound when found in aqueous media [20]. This is particularly problematic for autohydrolysis, as over 2.5 wt% phosphorus existed in ash derived from corn stover [21]. The remaining phosphorus would be released in the form of phosphate which could further influence lignocellulosic autohydrolysis. However, apart from phosphorus-providing fertilizers, around 2.0–2.8 wt% organic matters normally existed in a fertile soil to increase the soil stability and humic acids also comprise a large fraction of the organic matter present in farmland soil [22]. Humic acid could also be used as soil conditioner because it could enhance the fertilizer efficiency, improve the quality of soil and so on [23]. The high levels of phenolate and carboxylate functional groups existed in humic acid and its negative surface charges contribute to its high ability to bind metal cations [24]. Humic acids also could be protonated due to its polyelectrolyte effect and heterogeneity. Therefore, using humic acid as soil conditioners normally combined with conventional fertilizers could exert strong buffering capacity through the mechanisms of metal cations liberations by resisting the pH change of the soil [25]. Meanwhile, humic acid also displays surfactant-like behavior because of its specific chemical structures. Non-cationic surfactants present during autohydrolysis pretreatment could capture some of the lignin released into the liquid phase by forming emulsions which finally resulted in more lignin removal from lignocellulose [26]. It could be hypothesized that the content of humic acid in soil exerts two possible effects upon autohydrolysis performance (1) buffering capacity, or (2) enhancing delignification of pretreated residue. Therefore, considering the better contributions to the soil buffering capacity of these two soil conditioners (sodium phosphate and sodium humate), we studied the effects of sodium phosphate and sodium humate as model compounds of soil conditioners to better grapple with the effects that EA-derived soluble salt and organic matter exert upon autohydrolysis efficiency.

## Results and discussion

### Roles of buffering components on autohydrolysate properties

**Fig. 1:**
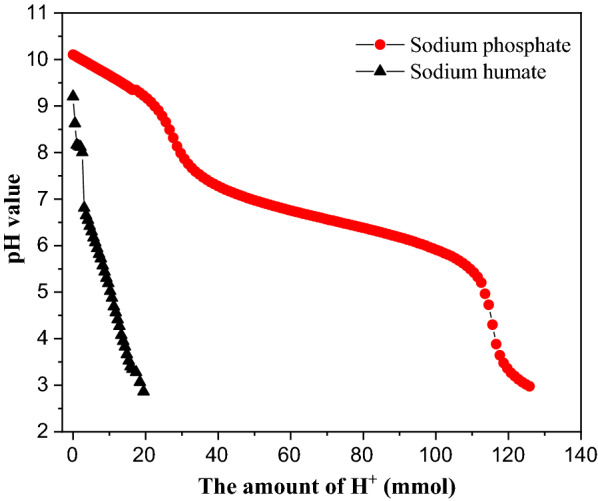
Acid titration curve of buffering model compounds (10 g/L)

Autohydrolysis of biomass has fostered considerable interests due to its weak acid medium without any other chemicals additions that can be imposed [27]. Normally, through autohydrolysis, xylan and lignin were fractionated which lead to an increase of cellulosic surface exposed to the cellulase. However, if buffering compounds existed in the process of lignocellulose autohydrolysis, the pretreatment efficiency might be restricted because of the reduction the H^+^ in the liquid. Based on the dilute acid titration results in Fig. 1, sodium phosphate exerted a much higher buffering capacity than sodium humate. When decreasing the pH from 6.5 to 3, sodium phosphate consumed 356.0 mmol H^+^ while sodium humate only consumed 19.4 mmol H^+^ at the same concentration (10 g/L). Therefore, it can be imagined that the same loadings of two different buffering compounds could result in various effects on the pretreatment efficiency. As shown in Table 1, increasing dosages (from 1 to 30 g/L) of sodium phosphate and sodium humate in WS autohydrolysis resulted in an increase to prehydrolysate pH values from 4.0 to 5.1 and from 4.1 to 4.7, respectively. According to our previous report, the pH of prehydrolysate generated without the presence of buffering compounds was 3.8 under the same pretreatment parameters [28]. Obviously, the added buffering compounds lead to a reduction to [H^+^] in the cooking medium. Importantly, we previously asserted that the acidity of the cooking results in different extents of lignocellulosic degradation by the pretreatment. But from the results regarding chemical composition of the prehydrolysate (Table 1), no changes to acetic acid concentrations in the prehydrolysate were detected regardless of the addition of buffering compounds. The extent of deacetylation during lignocellulose autohydrolysis was tightly related to the pretreatment parameters (temperature and cooking time) [29]. In this work, the autohydrolysis parameters were maintained at same level. Furthermore, similar results were also observed in another report in the literature [30].

**Table 1:** The pH values after pretreatment, fermentation inhibitors, monosaccharides concentrations and xylo-oligosaccharides in the prehydrolysate at different pretreated conditions

Additives	Concentration (g/L)	pH after the pretreatment	Fermentation inhibitors (g/L)				Monosaccharides concentration (g/L)			Xylo-oligosaccharide concentration (g/L)
			Formic acid	Acetic acid	Furfural	HMF^a^	Glucose	Xylose	Arabinose	
Na_3_PO_4_	1	4.0	0.5	1.8	0.0	0.0	0.1	2.2	0.5	8.6
	5	4.1	0.6	1.9	0.0	0.0	0.2	1.8	0.4	8.0
	10	4.3	0.7	2.0	0.0	0.0	0.0	1.1	0.2	6.9
	20	4.5	1.0	2.4	0.0	0.0	0.0	0.6	0.2	6.0
	30	5.1	1.0	2.4	0.0	0.0	0.0	0.4	0.2	4.9
Sodium humate	1	4.1	0.4	1.8	0.0	0.0	0.2	2.8	0.4	5.6
	5	4.1	0.2	2.1	0.0	0.0	0.2	2.9	0.4	4.4
	10	4.2	0.1	2.0	0.0	0.1	0.1	2.4	0.4	4.4
	20	4.5	0.1	2.1	0.1	0.0	0.1	1.9	0.2	4.0
	30	4.7	0.0	2.1	0.0	0.0	0.1	1.6	0.2	2.8

^a^Refers to hydroxymethylfurfural

Meanwhile, we also found that no HMF and furfural were detected in the prehydrolysate in the presence of the buffering components. This is not surprising, given that their generation is dependent upon acid-catalyzed dehydration of monosaccharides. Furthermore, these monosaccharides can only be formed from acid hydrolysis of hemicellulosic oligosaccharides [31, 32]. Therefore, a lessened extent of acidity results in lesser formation of all these components. Specifically, increasing loadings (from 1 to 30 g/L) of sodium phosphate and sodium humate caused the concentrations of xylose to be decreased from 2.2 to 0.4 g/L and from 2.8 to 1.6 g/L, respectively. It was also observed that the xylo-oligosaccharide concentrations also decreased from 8.6 to 4.9 g/L and from 5.6 to 2.8 g/L, respectively. To further understand the effects of the buffering by phosphate and humate, additional properties of prehydrolysate were analyzed in addition to the chemical composition of pretreated residues.

### Effects of additional buffering compounds in WS autohydorlysis on the prehydrolysate physical characterization and the chemical composition of pretreated WS

A significant amount of xylan (up to 80 wt% ) could be removed from WS under the tested pretreatment conditions (180 °C, 40 min). However, we have already shown from the results in Table 1 that things change when the tested buffering compounds are added during autohydrolysis. From Table 2, the percentage of xylan removal deceased from 84.3 to 61.4% and from 72.3 to 53.0% with increasing dosages of sodium phosphate and sodium humate (from 1 to 30 g/L; respectively). These results matched the minor concentrations of degradation products in the prehydrolysate we observed. In general, the amount of H^+^ in similar solvent conditions is clearly the key index for autohydrolysis performance. However, the properties of the cooking liquor might be changed by the loadings of buffering compounds.

**Table 2:** Effects of buffering compounds on the chemical compositions of pretreated WS

Additives	Concentration (g/L)	Recovery yield (%)		Removal yield (%)	
		Solid	Glucan	Xylan	Lignin
Na_3_PO_4_	1	59.6	87.6	84.3	20.2
	5	61.4	89.1	83.5	22.0
	10	61.2	90.5	80.4	23.3
	20	61.9	92.2	70.6	19.9
	30	70.9	94.3	61.4	18.1
Sodium humate	1	58.8	88.6	72.3	18.6
	5	59.4	85.5	70.9	31.7
	10	59.6	86.4	69.1	43.7
	20	61.2	87.2	64.5	41.8
	30	61.6	89.8	53.0	38.1

**Fig. 2:**
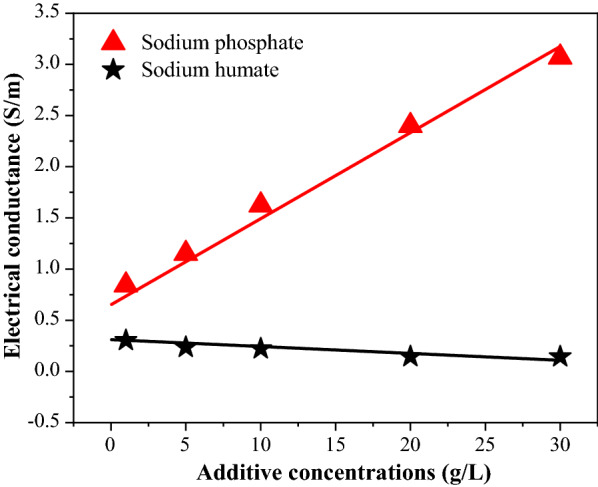
Electric conductivity of the prehydrolysate after pretreatment

The activities of hydronium ions (H^+^) in liquid are normally enhanced when the solution has high ionic strength, a parameter that can be measured through electric conductivity. As shown in Fig. 2A, the electric conductivities of the sodium phosphate-containing autohydrolysate increased from 0.8 to 3.1 S/m. Only minor changes of electric conductivities (ranging from 0.3 to 0.1 S/m) were found for the sodium humate autohydrolysate. This difference could be explained by the fact that humic acid as a polyelectrolyte is fundamentally different from the salt (sodium phosphate) which are comparing it against. Specifically, it is possible that the sodium humate could absorb H^+^ in the liquid based on its incomplete dissociation properties. Therefore, the activity of H^+^ in the prehydrolysate of sodium humate might be lower than that of sodium phosphate due to its low ionic strength and minimal contribution to solution conductivity [33].

As shown in Table 2, enhanced delignification (ranging from 18.6 to 43.7%) was achieved by additions of sodium humate, however no significant change to delignification (ranging from 18.1 to 23.3%) was observed for sodium phosphate. It has been reported that lignin removal from lignocellulose in autohydrolysis is tightly correlated to elevated temperatures [34]. Previous researches have pointed that parts of lignin melt at high temperature and further degrade to other products which eventually are solubilized into prehydrolysate. Humic acid itself was considered as a natural organic surfactant (contains aromatic rings) and its chemical structure can be quite similar with lignin [35]. Researchers have mentioned that humic acid could be used as the substitutes for conventional surfactants in some applications [36]. Therefore, it can be imagined the melted lignin from lignocellulose during autohydrolysis might be further extracted by the sodium humate acting as a surfactant in the system.

Finally, we observed similar levels of glucan recovery (above 85 wt% ) despite variance in xylan removal and delignification. This observation was attributed to the chemical stability of cellulose at the autohydrolysis conditions tested [37]. However, we do expect that enzymatic digestibility will be changed based on the already discussed differences in removal of xylan and lignin.

### Effects of loading buffering compounds on the enzymatic digestibility of pretreated WS

**Fig. 3:**
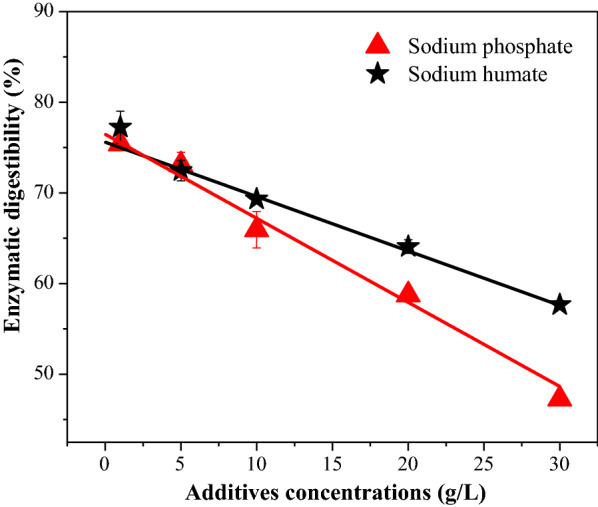
Effects of buffering compounds added in wheat straw autohydrolysis on the 72-h enzymatic digestibility of pretreated residue

Enzymatic hydrolysis results were no doubt the key rings which could be used to evaluate one pretreatment efficiency [38]. Figure 3 displays the effects of buffering compounds on the 72-h enzymatic digestibility of the pretreated residues. As shown in Fig. 3, with the increasing dosages (from 1 to 30 g/L) of sodium phosphate and sodium humate, the enzymatic digestibility of pretreated residues decreased from 75.4 to 47.3% and from 77.3 to 57.7%, respectively. Interestingly, linear relationships of *y* = 76.5 − 0.9x and *y* = 75.4 − 0.6x were recorded between the added amounts of sodium phosphate and sodium humate with the enzymatic digestibility of pretreated residues, respectively. Previous results indicated the increasing loadings of EA in WS autohydrolysis could result in a decrease to xylan removal of pretreated residues, which penalized downstream enzymatic digestibility [28]. Residual xylan in pretreated residues has been described to exert a “blocking effect” upon the substrate’s surface, which renders unsuccessful enzymatic digestion [39]. To negate or eliminate the unwanted buffering effects caused by ash during autohydrolysis, strategies of de-washing and addition of sulfuric acid and exchangeable metal salts have been successfully applied in previous work [21, 40, 41]. Previous studies have also observed that the removal of organic matter alone from the soluble mixtures of EA can lead to an increasing enzymatic digestibility of autohydrolyzed WS with EA components [28]. If part of sodium phosphate is replaced by sodium humate of the same quality and applied to WS autohydrolysis, the enhancement for enzymatic digestibility of autohydrolyzed WS will be reduced due to the reductions of buffering effects and certain amounts of delignification during autohydrolysis process, compared to pretreatment with sodium phosphate alone. However, even all the 30 g/L sodium phosphate was replaced by sodium humate, the 72-h enzymatic digestibility of autohydrolyzed WS was only 57.7%. Therefore, it could be speculated the WS autohydrolysis efficiency would be also hugely restricted by the co-existence of sodium phosphate and sodium humate. For autohydrolysis, significant amounts of xylan removal from lignocellulose exposes more accessible area of pretreated residues for enzymatic attack [42]. It was also reported that in the pretreatment of corn stover, the extent of xylan removal is more critical for establishing strong enzymatic digestibility compared to delignification [43]. According to the extent of xylan removal, it seemed the enzymatic digestibility of pretreated residues with 30 g/L sodium humate addition in pretreatment should be lower than that of 30 g/L sodium phosphate addition. However, the truth was opposite. In general, we also found that relatively higher amounts of lignin were removed in the presence of sodium humate versus sodium phosphate humate. It is widely agreed upon that lignin removal is also beneficial towards cellulolytic hydrolysis of pretreated lignocellulose by reducing non-productive adsorption between enzyme and residual lignin [44, 45]. Therefore, despite the dosages of sodium humate create a huge obstacle for xylan removal from lignocellulose, attributing its effects on lignin removal, the morphological properties of pretreated WS might be changed which finally resulted in higher enzymatic digestibility of pretreated WS than that of sodium phosphate [46]. To better explain the changes of pretreated residues enzymatic digestibility, the enzymatic accessibility was estimated using a dye adsorption assay.

### The relationship between cellulase accessibility and enzymatic digestibility of pretreated WS

**Fig. 4:**
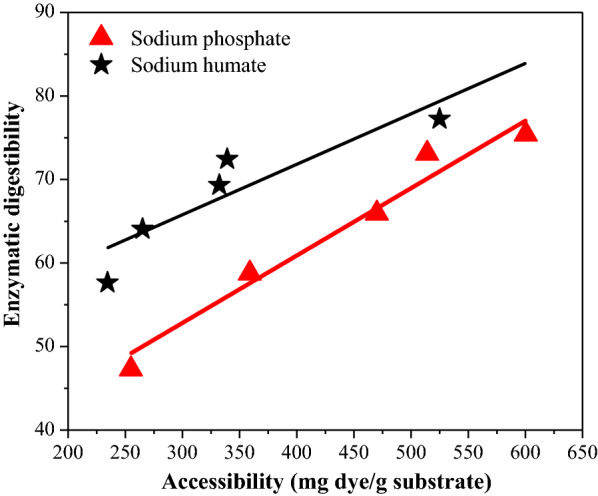
Relationships between enzymatic accessibility and digestibility of pretreated residues

Dye adsorption test has been successfully applied to estimate the enzymatic accessibility of pretreated residues. Furthermore, its results have been shown to correlate well with enzymatic hydrolysis outcome [47]. As shown in Fig. 4, an increase of enzymatic accessibility of pretreated residues could lead to an increase in enzymatic digestibility. As we previously pointed out, the unwanted buffering effects caused by EA in WS autohydrolysis lead to the decreasing enzymatic accessibility digestibility [28]. Similar trends were also observed while model buffering compounds of soil conditioners were dosed into WS autohydrolysis. Obviously, attributing to the model buffering compounds involved in WS autohydrolysis, the lesser extent of xylan removal is partially to blame for the decline in enzymatic accessibility for the pretreated residue. As shown in Fig. 4, increasing dosages (from 1 to 30 g/L) of sodium phosphate and sodium humate resulted in accessibilities decreasing from 600.4 to 255.0 mg dye/g substrate and from 525.0 to 234.5 mg dye/g substrate, respectively. The dosage response is most likely due to their strong buffering capacities. Despite the possibility of sodium humate increasing delignification, these results showed that its self-buffering capacity provided more of a negative influence upon digestibility compared to the possible benefit of improved delignification. The results summarized the different extent changes of pretreated WS accessibility which were mainly attributed to the existence of various buffering compounds in WS autohydrolysis and the accessibility was highly related to the efficiency of enzymatic digestibility.

## Conclusion

Autohydrolysis efficiency was found to be significantly reduced by the loadings of buffering compounds. The two compounds provided model systems that were found to prevent [H^+^] from reaching the desired levels, leading to a decrease in xylan removal. This was consistent across both sodium humate and sodium phosphate. Furthermore, the decrease in xylan removal also translated to low enzymatic digestibility. This was largely an issue of substrate accessibility, as revealed by a dye adsorption assay. Interestingly, improvement of delignification by loading sodium humate did not translate to any improvement in digestibility given the buffering effects of sodium humate. Therefore, our results show that two model compounds of soil conditioner that is often included in lignocellulosic crop residues also could buffer autohydrolysis media. These findings should guide future work looking to curb this buffering effect.

## Methods

### Materials and reagents

Wheat straw (WS) was obtained from a local farmer in Shandong province, China. Raw WS was treated by the reported procedures in our previous work to remove self-attached EA [28].

Sodium humate derived from lignite was purchased from Aladdin Reagent (Shanghai, China). The cellulase (Cellic^®^ CTec2) used in this work was purchased from Novozymes (NA, Franklinton, USA) with a measured filter paper activity of 250.0 FPU/mL. All remaining solvents, chemicals and reagents were of at least analytical grade.

### Autohydrolysis of WS

Autohydrolysis pretreatment was performed in a laboratory-customized oil bath reactor. Prior to pretreatment, the pH of the formulated phosphate and sodium humate solutions was reduced to pH 6.5 through addition of concentrated hydrochloric acid, respectively. For pretreatment, the reactor was loaded with 50 g (based on dry weight) of WS. In addition, varying concentrations (~ 1–30 g/L) of either phosphate or sodium humate solutions were loaded at solid to liquid ratio 1:10 (w/v). The vessel temperature was raised from 60 to 180 °C at a heating rate of 2 °C/min, and then held at the maximum temperature for 40 min. Pretreatment parameters were selected based on available literature [16, 29]. When pretreatment was completed, the reactor was immediately transferred from the oil bath to an ice water bath. After cooling down the reactor to room temperature, cloth bags were used to separate pretreated residues from liquid prehydrolysate. Finally, the pretreated solid residues were washed using distilled water until the wash water was neutral, and the pretreated residues were stored in sealed plastic bags at 4 °C for 48 h to equilibrate moisture.

### Dilute acid titration of additional buffering compounds

0.5 g sodium phosphate or sodium humate was dissolved with 50 mL de-ionized water with a stirring bar at the speed of 180 rpm, 50 °C for 1 h, respectively. After mixing well, 0.1 M H_2_SO_4_ was applied to titrate the mixture at a speed of 0.5 mL/min that was actively being agitated at 150 rpm. A multi-water quality meter (MM-60R, TOADKK, Tokyo, Japan) equipped with glass composite electrodes was used to record pH change. The terminal pH value was set at 3.0 and the pH value in the liquid of testing sample was automatically recorded every 30 s.

### Electric conductivity determinations of the WS autohydrolysate

To measure the electric conductivity of the prehydrolysate, 30 mL prehydrolysate was centrifuged at 8000 rpm for 10 min and then the supernatant was poured into a clean 50-mL beaker. Next, a conductivity meter was lowered into the solution. Then stable results were recorded.

### Enzymatic hydrolysis of autohydrolysis-pretreated WS

Enzymatic hydrolysis was performed in 125-mL screw-capped glass bottles. Hydrolysis was conducted at a substrate loading of 5% (w/v) of pretreated residues in a 50 mM citric acid buffer (pH = 4.8) with an enzyme dosing of 25 FPU/g-glucan. 0.2 mL of 10 g/L tetracycline was added to avoid microbial contamination and the total volume of enzymatic hydrolysis was 50 mL. The reaction was conducted at 50 °C using a thermostat shaker at 150 rpm for slurry agitation in an environmental incubator shaker (New Brunswick Scientific, USA). After 72 h of hydrolysis, hydrolysate aliquots were withdrawn for further analysis. The obtained enzymatic hydrolysate was centrifuged at 10,000 rpm for 10 min to remove suspended solids. Supernatant was then diluted in order to analyze monosaccharide concentrations by high-performance liquid chromatography (HPLC). The enzymatic digestibility of pretreated residue was calculated as the following equation:

$${\text{Enzymatic digestibility }}\left( {\text{\% }} \right) = \frac{\text{glucose in enzymatic hydrolysate (g)}}{{{\text{initial glucan in pretreated solid (g)}} \times 1. 1 1}} \times 1 0 0 {\text{\% }}$$

### Direct red staining of pretreated WS for accessibility estimation

The accessibility of pretreated residues was estimated by the direct red 28 (DR 28) staining assay according to literature [48]. For direct red adsorption, 1% (w/v) washed substrate was loaded into 50 mL bottle and mixed with 20 mL distilled water in which direct red dye was dissolved. A series of increasing DR 28 concentrations (0, 0.05, 0.1, 0.5, 1.0, 2.0, 3.0, 4.0 g/L) was applied into each bottle and the bottles were incubated in a rotary shaker at 60 °C and 150 rpm agitation for 24 h. After incubation, 10 mL was withdrawn and centrifuged at 2500 rpm for 5 min to remove suspended solids. Supernatant absorbance of samples was measured by UV–Vis spectrophotometry at 498 nm. According to the absorbance readings, the amount of adsorbed dye was calculated as the difference between initial dye concentration and the dye concentration in the supernatant. Langmuir non-linear regression was used to estimate the maximum adsorption capacity of direct red unto pretreated samples, which was interpreted as the enzymatic accessibility to pretreated residue.

### Chemical composition analysis of pretreated WS

All experiments were carried out in duplicate and the means of duplicate analyses was reported in each figure and table. The infrared moisture meter (FD-720, KETT) was used to determine the moisture content of the pretreated materials. Chemical compositional analyses of all pretreated materials and prehydrolysates were carried out following the procedures of the NREL (National Renewable Energy Laboratory, Golden, Co, USA) standard analysis methods [49]. Determination of xylo-oligosaccharides was performed by 4 wt% H_2_SO_4_ hydrolysis at 121 °C for 60 min according to the literature reported procedures [50]. Xylo-oligosaccharide concentrations were calculated from the increase in xylose concentration before and after acid hydrolysis. Analysis of monosaccharides and inhibitors was performed using HPLC (Agilent 1260 series, Agilent Technologies, Santa Clara, CA, USA). with an Aminex HPX-87H column and a refractive index (RI) detector. The column temperature was maintained at 55 °C and the mobile phase was 0.005 mol/L H_2_SO_4_ at a flow rate of 0.6 mL/min.

## Data Availability

All data generated and analyzed in this study are included in this published article.

## Abbreviations

EA: Exogenous ash
WS: Wheat straw
NREL: National Renewable Energy Laboratory
HPLC: High-performance liquid chromatography
HMF: Hydroxymethylfurfural
